# Functional Characterization of Duplicated *SUPPRESSOR OF OVEREXPRESSION OF CONSTANS 1*-Like Genes in Petunia

**DOI:** 10.1371/journal.pone.0096108

**Published:** 2014-05-01

**Authors:** Jill C. Preston, Stacy A. Jorgensen, Suryatapa G. Jha

**Affiliations:** Department of Plant Biology, The University of Vermont, Burlington, Vermont, United States of America; Universidad Miguel Hernández de Elche, Spain

## Abstract

Flowering time is strictly controlled by a combination of internal and external signals that match seed set with favorable environmental conditions. In the model plant species *Arabidopsis thaliana* (Brassicaceae), many of the genes underlying development and evolution of flowering have been discovered. However, much remains unknown about how conserved the flowering gene networks are in plants with different growth habits, gene duplication histories, and distributions. Here we functionally characterize three homologs of the flowering gene *SUPPRESSOR OF OVEREXPRESSION OF CONSTANS 1* (*SOC1*) in the short-lived perennial *Petunia hybrida* (petunia, Solanaceae). Similar to *A. thaliana soc1* mutants, co-silencing of duplicated petunia *SOC1*-like genes results in late flowering. This phenotype is most severe when all three *SOC1*-like genes are silenced. Furthermore, expression levels of the *SOC1*-like genes *UNSHAVEN* (*UNS*) and *FLORAL BINDING PROTEIN 21* (*FBP21*), but not *FBP28*, are positively correlated with developmental age. In contrast to *A. thaliana*, petunia *SOC1*-like gene expression did not increase with longer photoperiods, and *FBP28* transcripts were actually more abundant under short days. Despite evidence of functional redundancy, differential spatio-temporal expression data suggest that *SOC1*-like genes might fine-tune petunia flowering in response to photoperiod and developmental stage. This likely resulted from modification of *SOC1*-like gene regulatory elements following recent duplication, and is a possible mechanism to ensure flowering under both inductive and non-inductive photoperiods.

## Introduction

Flowering time is a complex trait shaped by internal and external signals that interplay to determine reproductive success [1], [2]. On the one hand selection acts against mutations that cause flowering to occur during sub-optimal times of the year, such as in drought, freezing temperatures, or when pollinator abundances are low. On the other hand, plants that rarely experience optimal conditions must chance flowering eventually, or find alternative reproductive strategies (e.g. clonal reproduction and apomixis). Thus, flowering time pathways have evolved to both respond to, and buffer against, environmental variation. These seemingly opposing forces can be achieved either by integrating signals from parallel genetic pathways or by differentially utilizing related genes within the same pathway.

In the annual rosid species *Arabidopsis thaliana* (Brassicaceae) long day induced flowering is controlled by CONSTANS (CO)-mediated upregulation of the integrator protein FLOWERING LOCUS T (FT) in leaves [3]–[5]. FT protein is translocated through the phloem to the shoot apical meristem (SAM) where it binds with FLOWERING LOCUS D (FD) to induce the expression of floral promoters such as *SUPPRESSOR OF OVEREXPRESSION OF CONSTANS 1* (*SOC1*), *APETALA1* (*AP1*), *FRUITFULL* (*FUL*), and *LEAFY* (*LFY*) [6]–[8]. Although largely functionally conserved across both long and short day-induced flowering plants [9]–[11] alterations of *SOC1*-like gene function have been implicated in flowering time and plant habit evolution [11]. For example, in perennial short day strawberry (*Fragaria vesca*, Rosaceae) SOC1 represses flowering through a novel regulatory interaction with the flowering repressor *TERMINAL FLOWER1* (*TFL1*) [12]. Thus, fine-scale characterization of *SOC1*-like genes in diverse species has great potential to illuminate our understanding of flowering phenology and its evolution.

**Figure 1:**
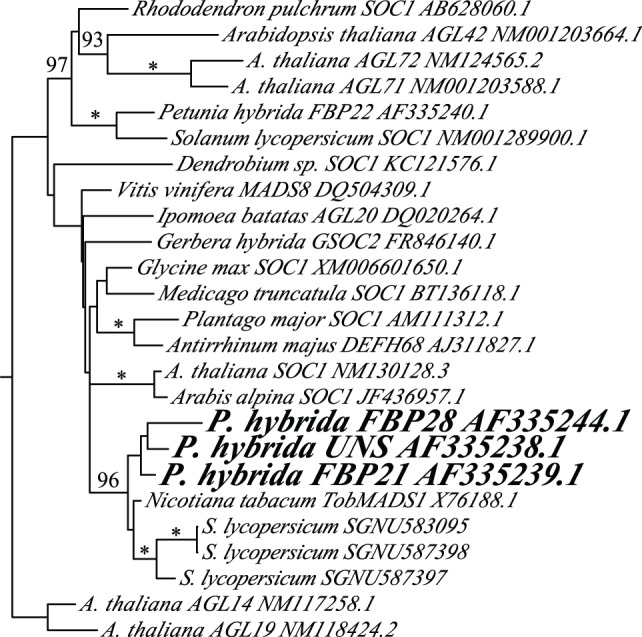
Maximum likelihood phylogeny of *SOC1*-like genes in petunia (bold) and other angiosperms. The tree is rooted on TM3 MADS-box genes in the *AGL14/19* clade sister to *SOC1/AGL42/AGL71/AGL72* genes [30]. Numbers indicate maximum likelihood bootstrap values, with 100% being represented by an asterisk. Sol Genomics Network (SGN) and Genbank accession numbers are shown after each gene name.

Petunia (*Petunia hybrida*, Solanaceae) is a short-lived perennial in the asterid lineage of core eudicots that is induced to flower by long day photoperiods [13]. The petunia genome contains three *SOC1* homologs – *UNSHAVEN (UNS)/FLORAL BINDING PROTEIN 20 (FBP20), FBP21,* and *FBP28* – derived from two duplication events within the Solanaceae (Fig. 1). All three genes are strongly expressed in leaves [14] and at least *UNS* is expressed in vegetative apices, with expression becoming reduced following the transition to flowering [15]. Null mutations in *UNS* and *FBP28* cause no obvious mutant phenotype [15], [16]. However, constitutive expression of *UNS* in petunia and *FBP21* in tobacco (*Solanum tabacum*) causes early flowering under short days, bract-like petals, and hairy ovaries [15], and accelerated flowering under long days, reduced plant height and leaf size, and increased flowering branches [17], respectively. The late flowering phenotype of both species suggests that *UNS* and *FBP21* promote the transition to flowering similar to *Arabidopsis thaliana SOC1* [18]. However, the relative importance of *UNS, FBP21*, and possibly *FBP28*, in flowering time regulation and plant architecture has yet to be rigorously tested.

Here we use virus-induced gene (co)-silencing (VIGS) and gene expression analyses to functionally characterize the petunia *SOC1*-like genes. Our data suggest a conserved role for all three genes in the promotion of flowering. However, the contribution of each gene to flowering likely varies under different photoperiods and in an age-dependent manner.

## Materials and Methods

### Plant Materials and Growth Conditions

Petunia ‘Fantasy Blue’ (2PET131) seed from Seedman.com were grown and selfed for at least two generations under standard greenhouse conditions. For the photoperiod experiment, plants were either grown under long day (16 h light: 8 dark) or short day (8 h light: 16 h dark) conditions at 22°C in controlled growth chambers. For VIGS experiments, plants were grown from seed in two batches at the University of Vermont greenhouse from September 2012 and August 2013 with supplemental light to mimic long days (16 h light: 8 h dark). Diurnal temperatures ranged from 20 to 24°C. Significant differences in days to flowering and leaf number at flowering were determined using the analysis of variance (aov) and posthoc Tukey test functions in R.

### Phylogenetic Analysis

*SOC1*-like gene sequences of representative angiosperms were downloaded from Genbank after a BLAST search with *SOC1* from *A. thaliana*. The closest *A. thaliana SOC1* paralogs *AGL14, AGL19, AGL42, AGL72,* and *AGL71* were also downloaded for reference and to root the tree. Amino acid sequences were aligned manually in Mesquite version 2.75 [19] (File S1). Nucleotide sequences were then subjected to maximum likelihood phylogenetic analysis in GARLI under a GTR+I+Γ model of evolution with 200 bootstrap replicates [20].

### Vector Construction and Plant Transformation

Previous results suggest functional redundancy of petunia *SOC1*-like genes under standard greenhouse conditions [15]–[17]. Thus, in order to target more than one petunia *SOC1*-like gene for silencing, but without affecting the expression of more distantly related paralogs, two VIGS constructs were designed based on approximately 200 bp sequences spanning part of the coiled-coil keratin-like and C-terminal domains of *FBP21* and *FBP28*. The *FBP21* region was 85, 68, and 40% identical to *UNS*, *FBP28*, and *FBP22* (next closest paralog), respectively; and the *FBP28* region was 83, 82, and 60% identical to *UNS*, *FBP21*, and *FBP22*, respectively. Gene fragments were amplified from petunia floral cDNA with gene specific primers containing restriction fragment ends (Table S1), sequence verified, and cloned into the tobacco rattle virus 2 (TRV2) vector. Each VIGS vector was transformed into *Agrobacterium tumefaciens* strain EHA105. A 194 bp fragment of the petal pigment gene *CHALCONE SYNTHASE* (*CHS*) was also amplified, cloned, and ligated into TRV2 for use as an experimental control as previously described [21]. Bacterial growth and plant infiltration methods followed Drea *et al.* (2007) and Hileman *et al.* (2005) [22], [23]. Twenty plants at the four-leaf pair stage for each silencing target were infiltrated in half their leaves with a 1∶1 ratio of TRV1 and TRV2 using a needleless syringe for a total of 60 plants (*CHS*-, *FBP21*-, and *FBP28*-TRV2). To target all three *SOC1*-like genes for silencing, a batch of 20 plants was also infiltrated with TRV1, *FBP21*-TRV2, and *FBP28*-TRV2 in a 2∶1∶1 ratio.

### Gene Expression and Phenotyping

RNA was extracted from leaves, SAMs, and nodes on the main stem of wild type plants at different developmental stages three hours post-zeitgeber, and in leaves at different times post-zeitgeber. Total RNA was extracted using TriReagent (Life Technologies) according to the manufacturer’s instructions, DNA was degraded using TURBO DNase (Life Technologies), and cDNA was synthesized using 1 µg RNA and iScript reverse transcriptase (BioRad). Primers for quantitative PCR (qPCR) of the housekeeping genes *EF1alpha* and *UBQ5, UNS, FBP21*, and *FBP28* were designed in Primer3 [24] and tested for efficiency using Fast SYBR Green Master Mix (Life Technologies) as previously described [25]. Primer pairs that gave high PCR efficiencies and single melt curves were selected for gene expression analyses (Table S1). After correcting for transcriptional activity, cycle threshold (C_T_) values were normalized against the geomean of the two housekeeping genes for three technical replicates and two to three biological replicates. To verify infection with TRV2 constructs and gene silencing, RNA was extracted from the tips of non-flowering branches (SAMs plus young leaves) in plants with the first flower at anthesis, and cDNA was synthesized. VIGS plants were screened for TRV2 using the primers pYL156F and pYL156R as previously described [23]. qPCR expression of *UNS, FBP21*, and *FBP28* was compared between ten plants positive for *FBP21*-TRV2 or *FBP28*-TRV2, and *CHS*-TRV2. Each VIGS positive plant was phenotyped for number of days from germination to anthesis, leaf number at anthesis, and petal whitening. Significant differences in gene expression were determined using the analysis of variance (aov) and posthoc Tukey test functions in R.

## Results and Discussion

### Petunia *SOC1*-like Genes are Partially Redundant in the Control of Flowering Time

**Figure 2:**
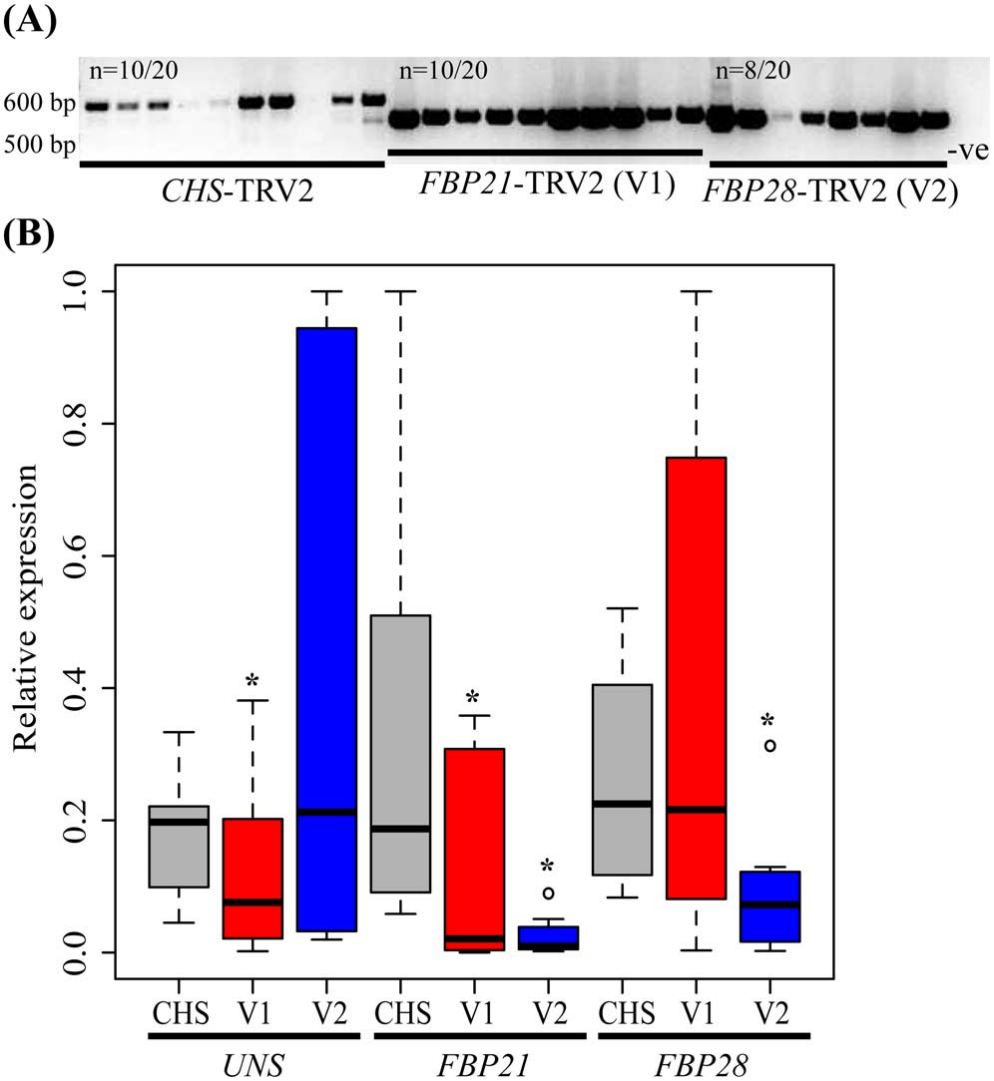
VIGS efficiency in petunia. (A) Typical agarose gel showing ethidium bromide stained amplicons using the TRV2-specific primers. Each lane represents a different individual in the VIGS experiment. −ve, negative control lacking cDNA. Of the 20 plants screened per treatment results for only 8 (*FBP28*-TRV2) or 10 (*CHS*-TRV2 and *FBP21*-TRV2) are shown as examples of efficiency. (B) Boxplots showing relative qPCR cT values for *UNS, FBP21*, and *FBP28* amplification. Each gene was amplified in ten plants infected with *CHS*-TRV2 (gray), *FBP21*-TRV2 (V1) (red), or *FBP28*-TRV2 (V2) (blue). Asterisks indicate at least a 2-fold average difference in gene expression between *FBP21*- or *FBP28*-TRV2 and control plants. Circles denote outliers.

Infiltration of petunia seedlings with *Agrobacterium* resulted in a total of 24, 21, 37, and 4 plants positive for the *CHS-, FBP21-, FBP28*-, and *FBP21/28*-TRV2 constructs, respectively (Fig. 2A). Relative expression analyses on vegetative SAMs of flowering individuals revealed significant silencing of *UNS* and *FBP21*, but not *FBP28*, by the *FBP21*-TRV2 construct (Fig. 2B). Although the *FBP28*-TRV2 construct had high sequence similarity to *UNS*, *FBP21*, and *FBP28*, only *FBP21* and *FBP28* were silenced using this construct. This disparity might be explained by the number of contiguous 21 bp matches between the three target *SOC1*-like genes, which is the minimum recognition length for RNA interference in plants. Thus, all three genes were targeted for silencing when plants were positive for *FBP21*- and *FBP28*-TRV2, and different combinations of two genes were silenced when positive for one construct alone.

**Figure 3:**
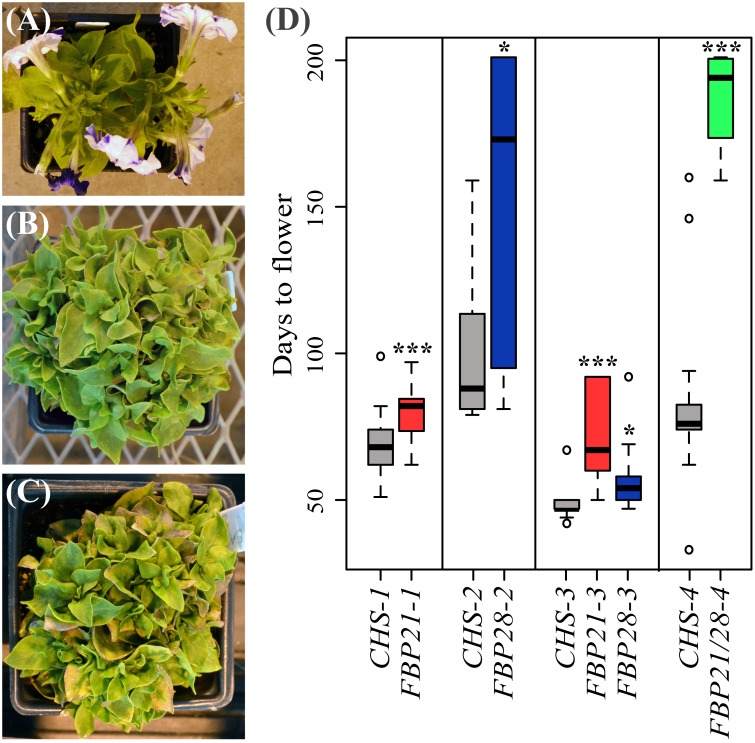
VIGS phenotypes. (A) Flowering *CHS*-TRV2 positive plants showing lack of anthocyanin pigment in normally purple petals. (B) Late flowering *FBP21*-TRV2 plant. (C) Later flowering *FBP28*-TRV2 plant. (D) Boxplots showing number of days to flowering for plants positive for *CHS*-TRV2 (gray), *FBP21*-TRV2 (red), *FBP28*-TRV2 (blue), or *FBP21*/*FBP28*-TRV2 (green) constructs. Vertical lines separate batches of plants that were grown at different times of the year. Circles denote outlier values. Three asterisks indicate p values <0.001 relative to control plants. A single asterisk indicates p values <0.05 relative to control plants.

In order to test the hypothesis that *SOC1*-like genes positively regulate flowering in petunia, days to flowering were calculated for each infected group of plants (Fig. 3). Data were compared between *CHS*-infected control plants, which all showed loss of anthocyanin accumulation in petals (Fig. 3A), and *SOC1*-like infected VIGS plants (Fig. 3B–E). The flowering response varied across plants as expected for incomplete and variable silencing, and between treatment blocks as predicted based on differences in ambient greenhouse light. Despite this, *SOC1*-like silenced plants in every category were later flowering on average than *CHS*-TRV2 control plants grown at the same time (Fig. 3D). The most extreme late flowering phenotypes were observed for the triple silenced plants positive for both *FBP21*- and *FBP28*-TRV2 (p<0.001). Moreover, plants silenced for *UNS* and *FBP21* (*FBP21*-TRV2 vector) were later flowering (p<0.001) than plants silenced for *FBP21* and *FBP28* (*FBP28*-TRV2 vector) (p = 0.015) when grown under the same conditions (Fig. 3D).

### Petunia *SOC1*-like Genes are Differentially Regulated by Photoperiod and Age

Heterogeneous protein function is often correlated with differences in the spatiotemporal pattern of underlying gene expression. In order to determine if petunia *SOC1*-like genes are differentially regulated diurnally, during development, and in response to different photoperiods, qPCR was conducted on wild type tissues. No significant differences in *SOC1*-like gene expression levels were found across 16 hours of daylight. This result is in contrast to *A. thaliana SOC1* expression, which peaks 12 hours post-zeitgeber in long day photoperiods [26]. Thus, petunia *SOC1*-like genes could potentially promote flowering in light under both long and short day conditions.

**Figure 4:**
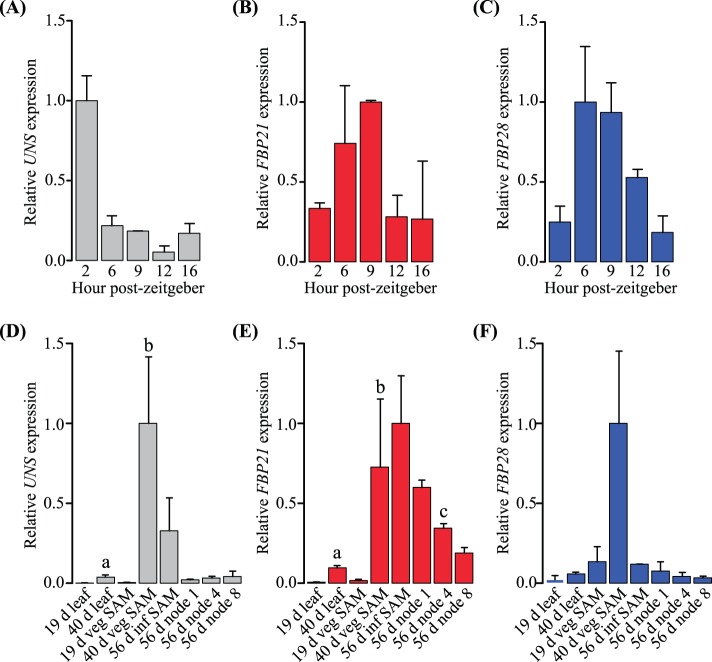
Relative expression of petunia *UNS* (gray), *FBP21* (red), and *FBP28* (blue). (A–C) Transcript levels of *SOC1*-like genes vary in the youngest (upper) leaves throughout 16 h long days relative to the zeitgeber (dawn), but these trends are not significant. (D–F) *SOC1*-like gene transcripts are most abundant in SAMs relative to leaves and nodes. However, the peak of expression in each tissue type varies between genes. Bars are averages for two to three biological replicates with standard deviations. Significant differences at the 0.05 level are denoted by letters: a, leaves; b, SAMs; and c, nodes.

Transcript levels of *UNS* and *FBP21* significantly increased in leaves (p<0.05 and <0.01, respectively) and SAMs (p<0.05 and <0.05, respectively) during vegetative development (Fig. 4D and 4E). However, expression was not significantly different for *FBP28*, or any of the *SOC1*-like genes between vegetative and inflorescence apices (Fig. 4D–F). With the exception of *FBP28*, these data are consistent with reports of *A. thaliana SOC1* expression [18], [27]. Following the transition to flowering at day 56, *FBP21* decreased significantly (p<0.001) in nodes from the plant base to the SAM (Fig. 4E). In contrast, no significant differences in nodes expression were detected for *UNS* and *FBP28* (Fig. 4D and 4F).

**Figure 5:**
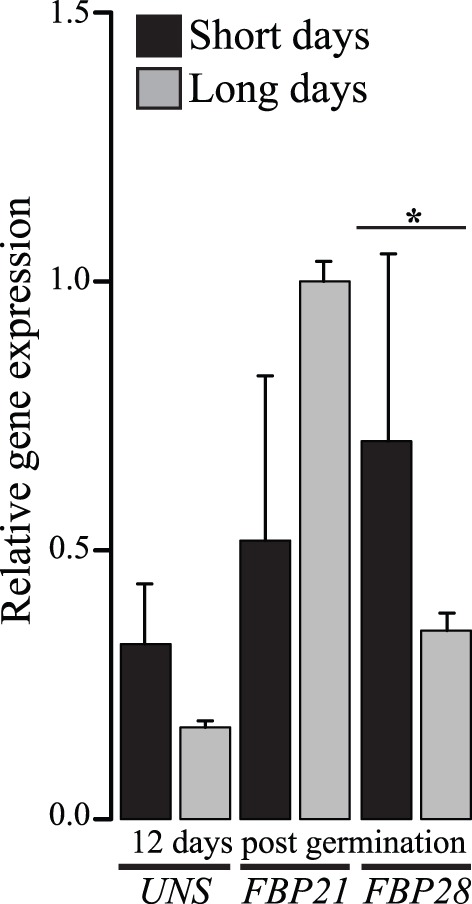
Relative leaf expression of *SOC1*-like genes in response to short (8 h light: 16 h dark) (black bars) versus long (16 h light: 8 h dark) (gray bars) days. Tissue was collected three hours post-zeitgeber under both photoperiod conditions. *FBP28*, but not *UNS* and *FBP21*, is more strongly expressed after 12 days grown under short versus long day conditions (p<0.05 denoted by an asterisk). Bars are averages for two to three biological replicates with standard deviations.

Although petunia is a long day plant, flowering earlier with increasing daylight hours, *FBP28* was significantly (p<0.05) more strongly expressed in the fully expanded top leaf 12 days post-germination when grown under short versus long days (Fig. 5). In contrast, there was no significant photoperiod effect on the expression of *UNS* and *FBP21* (Fig. 5). Taken together, data from this experiment suggest subtle differences in the spatiotemporal regulation of petunia *SOC1*-like genes during vegetative development and by photoperiod, with *FBP28* expression being the most divergent compared to *A. thaliana SOC1* [18], [27]. We hypothesize that *FBP28* has been recruited to ensure flowering under short days in petunia, and that the regulatory elements of all three *SOC1*-like genes have evolved following their recent duplication.

## Conclusions

Our data support the hypothesis that petunia *SOC1*-like genes have retained their function as flowering time pathway integrators following duplication [15], [17]. This is in contrast to the closely related *SOC1*-like gene *GhSOC1* in *Gerbera hybrida* (Asteraceae) that appears only to function in late flower development [28]. However, differential expression of *UNS, FBP21*, and *FBP28* in response to photoperiod and age suggests subtly different developmental roles to both ensure and fine-tune flowering under variable environmental conditions. A similar mechanism has been assigned to duplicated *FLOWERING LOCUS C* (*FLC*)/*MADS AFFECTING FLOWERING* (*MAF*) genes in *A. thaliana* that response to different low and high temperature cues [29]. Future studies determining the effect of co-silencing *SOC1*-like genes under variable photoperiod and temperature conditions will be needed to further test this hypothesis.

## Supporting Information

File S1 *SOC1*-like gene nucleotide alignment.
(NEX)

Table S1 Primer pairs used for VIGS and qPCR.
(DOCX)
