# Supplementary material for: Functional Characterization of Duplicated SUPPRESSOR OF OVEREXPRESSION OF CONSTANS 1-Like Genes in Petunia
Source: PLoS One. 2014 May 1;9(5):e96108. doi: 10.1371/journal.pone.0096108 (PMC4006870; doi:10.1371/journal.pone.0096108)
Supplement: Table S1 — Primer pairs used for VIGS and qPCR. (DOCX) [file pone.0096108.s002.docx]

**Table 1**. Petunia primers

| **Primers** | **Sequence (5’-3’)** | **Primer pair** |
| --- | --- | --- |
| *VIGS primers* |  |  |
| FBP21.BamHI.F | ACAGGATCCATCCTTGCTGCTGAAAATGC | FBP21.XhoI.R |
| FBP21.XhoI.R | ACACTCGAGCTCAAGCTATGCATCCAACG | FBP21.BamHI.F |
| FBP28.BamHI.F | ACAGGATCCGACGCAGCAAGTCTGATGAA | FBP28.XhoI.R |
| FBP28.XhoI.R | ACACTCGAGCCACAGCATTGCATTTTCAG | FBP28.BamHI.F |
|  |  |  |
| *qPCR primers* |  |  |
| PhEF1alpha.F | TGTTCTCTGCCTTGTATGTCTGG | PhEF1alpha.R |
| PhEF1alpha.R | TCAAAAGAGGCAGGCAGACAG | PhEF1alpha.F |
| PhUBQ5.F | TGGAGGATGGAAGGACTTTGG | PhUBQ5.R |
| PhUBQ5.R | CAGGACGACAACAAGCAACAG | PhUBQ5.F |
| PhUNS.q.F | ACGGAGTGTCAGCATCATCA | PhUNS.R |
| PhUNS.q.R | TGCACTCAGGTGGTCCAAA | PhUNS.F |
| PhFBP21.q.F | CTCCAAACGCAGAAATGGAT | PhFBP21.R |
| PhFBP21.q.R | TGTTCCCCAGCTTGATTTTC | PhFBP21.F |
| PhFBP28.q.F | CTTGACGCAGCAAGTCTGAT | PhFBP28.R |
| PhFBP28.q.R | TTCTTGCACGGATAGTGCTG | PhFBP28.F |
